# Disruption of cardiac cholinergic neurons enhances susceptibility to ventricular arrhythmias

**DOI:** 10.1038/ncomms14155

**Published:** 2017-01-27

**Authors:** Christiane Jungen, Katharina Scherschel, Christian Eickholt, Pawel Kuklik, Niklas Klatt, Nadja Bork, Tim Salzbrunn, Fares Alken, Stephan Angendohr, Christiane Klene, Janos Mester, Nikolaj Klöcker, Marieke W. Veldkamp, Udo Schumacher, Stephan Willems, Viacheslav O. Nikolaev, Christian Meyer

**Affiliations:** 1 Department of Cardiology-Electrophysiology, cardiac Neuro- and Electrophysiology Research Group (cNEP), University Heart Center, University Hospital Hamburg-Eppendorf, Martinistrasse 52, 20246 Hamburg, Germany; 2 DZHK (German Center for Cardiovascular Research), partner site Hamburg/Kiel/Lübeck, 13347 Berlin, Germany; 3 Institute of Experimental Cardiovascular Research, University Medical Center Hamburg-Eppendorf, Martinistrasse 52, 20246 Hamburg, Germany; 4 Department of Nuclear Medicine, University Medical Center Hamburg-Eppendorf, Martinistrasse 52, 20246 Hamburg, Germany; 5 Institute of Neural and Sensory Physiology, Medical Faculty, University of Düsseldorf, Universitätsstrasse 1, 40225 Düsseldorf, Germany; 6 Academic Medical Center, University of Amsterdam, Department of Clinical and Experimental Cardiology, Meibergdreef 9, 1105 AZ Amsterdam, The Netherlands; 7 Institute of Anatomy and Experimental Morphology, University Cancer Center Hamburg, University Medical Center Hamburg-Eppendorf, Martinistrasse 52, 20246 Hamburg, Germany

## Abstract

The parasympathetic nervous system plays an important role in the pathophysiology of atrial fibrillation. Catheter ablation, a minimally invasive procedure deactivating abnormal firing cardiac tissue, is increasingly becoming the therapy of choice for atrial fibrillation. This is inevitably associated with the obliteration of cardiac cholinergic neurons. However, the impact on ventricular electrophysiology is unclear. Here we show that cardiac cholinergic neurons modulate ventricular electrophysiology. Mechanical disruption or pharmacological blockade of parasympathetic innervation shortens ventricular refractory periods, increases the incidence of ventricular arrhythmia and decreases ventricular cAMP levels in murine hearts. Immunohistochemistry confirmed ventricular cholinergic innervation, revealing parasympathetic fibres running from the atria to the ventricles parallel to sympathetic fibres. In humans, catheter ablation of atrial fibrillation, which is accompanied by accidental parasympathetic and concomitant sympathetic denervation, raises the burden of premature ventricular complexes. In summary, our results demonstrate an influence of cardiac cholinergic neurons on the regulation of ventricular function and arrhythmogenesis.

## Abstract (web summary)

Catheter ablation is a common therapy for atrial fibrillation but disrupts cardiac cholinergic neurons. Here the authors report that cholinergic neurons innervate heart ventricles and show that their ablation leads to increased susceptibility to ventricular arrhythmias in mouse models and in patients.

The autonomic nervous system of the heart and its impact on heart rate and rhythm are known for more than a century^1^. Recent advances demonstrate the importance of the cardiac neural network in the pathophysiology of atrial fibrillation (AF). This is the most common sustained human arrhythmia, with a rising incidence of up to 10% in octogenarians, and leads to significant morbidity and mortality^2^,^3^. Cardiac neurons are inevitably obliterated during catheter ablation^4^, which is the therapy of choice for most patients suffering from symptomatic AF. This technique is a minimally invasive procedure performed to deactivate specific areas of abnormal firing heart tissue especially in the left atrium around the pulmonary veins. As the atrial neural network contains mostly cholinergic neurons, an increase in heart rate after catheter ablation is widely recognized to result from this partial atrial denervation (PAD)^2^. Although PAD during catheter ablation of AF seems to alter atrial electrophysiology beneficially^4^,^5^, its impact on ventricular electrophysiology remains unclear.

Text book knowledge states that parasympathetic axonal supply is limited to the atria^6^, but this paradigm has recently been challenged^7^,^8^. There is some evidence supporting the notion that extracardiac parasympathetic efferents are able to modulate ventricular function^9^,^10^ and electrophysiology^11^ by involving central and peripheral neural pathways^12^,^13^,^14^. In addition, cardiac ganglia have been shown to influence ventricular repolarization properties^15^. However, the relevance of cardiac cholinergic neurons for ventricular electrophysiology is not yet understood.

Here, we present clinical data demonstrating impaired ventricular electrophysiology following catheter ablation in patients. Experimental data confirm that mechanical disruption or pharmacological blockade of cardiac parasympathetic innervation shortens ventricular refractory periods (VRPs), increases the incidence of ventricular arrhythmia and decreases ventricular cAMP levels in mouse hearts. Confirmingly, immunohistochemical stainings show that cardiac cholinergic nerve fibres run from the atria to the ventricles in parallel to sympathetic fibres. The results imply a vital role of atrial cholinergic neurons in the modulation of ventricular electrophysiology.

## Results

### PAD decreases ventricular refractory periods

**Figure 1:**
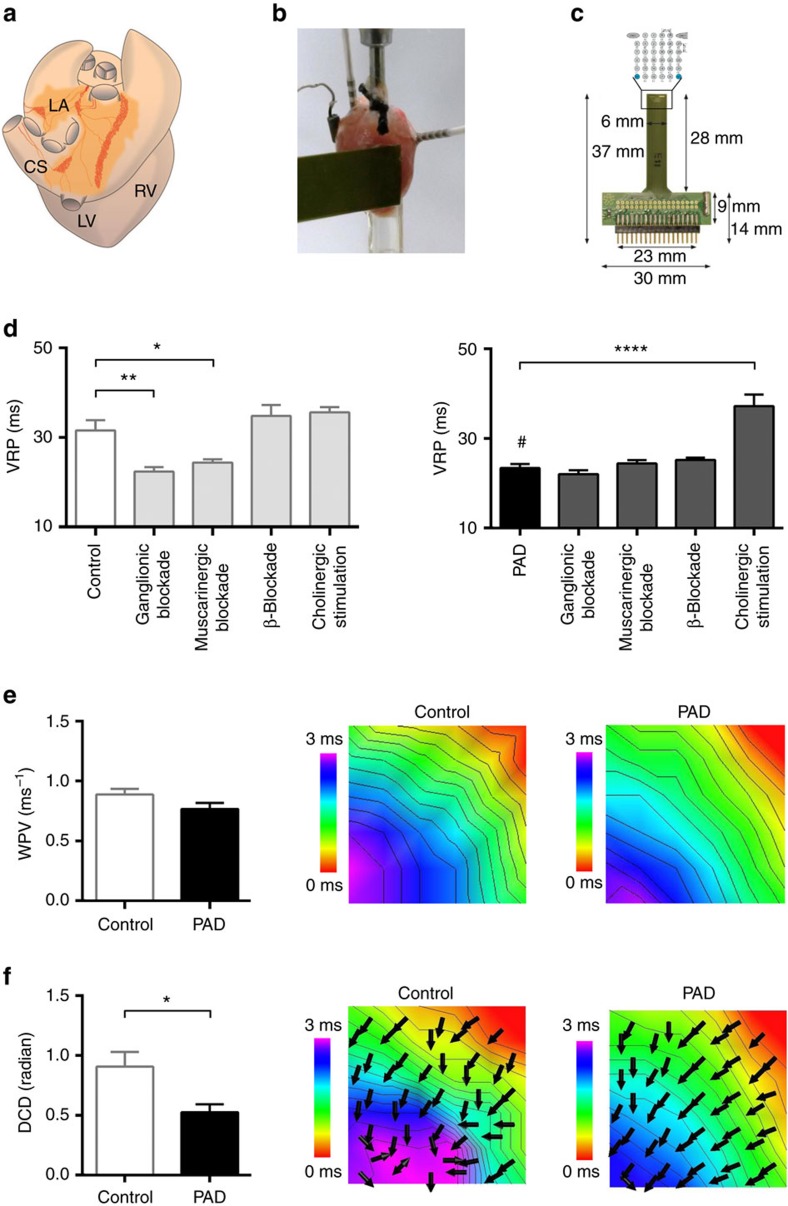
Disruption of atrial cholinergic control modifies ventricular electrophysiology. (**a**) Schematic drawing of a murine heart in posterior view. Light and dark orange surfaces depict areas of atrial fat with ganglionated plexi, which have been mechanically removed in partial atrial denervation (PAD) experiments. CS, coronary sinus; LA, left atrium; LV, left ventricle; RV, right ventricle. (**b**) Example of a murine heart within the Langendorff setup. Intracardial and epicardial catheters, as well as the epicardial multi-electrode array, are depicted. (**c**) The epicardial multi-electrode array is depicted with an enlargement of the schematic electrode layout. (**d**) The ventricular refractory periods (VRPs) are presented for control and PAD hearts with different pharmacological interventions. In comparison with control (*n*=10), the VRP was decreased by PAD (denoted by #; *n*=10; *P*=0.003; unpaired *t*-test), by ganglionic blockade (hexamethonium, 5 × 10^−4^ M; *n*=5; *P*=0.002; Mann–Whitney test) and by muscarinergic blockade (atropine, 1 × 10^−5^ M; *n*=5; *P*=0.045; unpaired *t*-test). β-Blockade (propranolol, 1 × 10^−6^ M; *n*=5) and cholinergic stimulation (acetylcholine, 1 × 10^−5^ M; *n*=5) did not influence VRPs in controls. After PAD ganglionic, muscarinergic or β-blockade did not reduce the VRP. However, cholinergic stimulation in PAD hearts raised the VRP (*n*=5; *P*<0.0001; unpaired *t*-test) suggesting reversibility of PAD-induced shortening of VRPs. (**e**) Wave propagation velocity (WPV) was not influenced by PAD as depicted in the colour-coded reconstruction with isochrones (2 m s^−1^ distance between isochrones) of epicardial multi-electrode activation mapping. (**f**) Dispersion of conduction direction (DCD) was smaller after PAD (*n*=16) compared with controls (*n*=19; *P=*0.026; Mann–Whitney test). Note the more homogeneous arrow alignment in the right image. All the values shown are mean±s.e.m. *P<0.05, **P<0.01, ****P<0.0001.

To investigate whether disruption of cardiac neurons influences baseline electrophysiology, we first performed electrophysiological studies in mouse hearts (Fig. 1a–f). We determined standard electrophysiological parameters including the VRP, which describes the duration of time in which the cardiac cell is unable to initiate another action potential. VRPs were shorter in PAD hearts (23.4±0.9 ms) compared with control hearts (31.6±2.2 ms, *P*=0.003, unpaired *t*-test; Fig. 1d), similar to atrial refractory periods (see Supplementary Table 1), demonstrating that disruption of cardiac neurons distant from the ventricles influences ventricular baseline electrophysiology. In control hearts, the VRPs were shortened by ganglionic (22.4±1.0 ms, *P*=0.002, Mann–Whitney test) or muscarinergic blockade (24.4±0.7 ms, *P*=0.045, unpaired *t*-test) but not altered by β-blockade (34.8±2.4 ms, *P*=0.391, unpaired *t*-test) or cholinergic stimulation (35.6±1.2 ms, *P*=0.248, unpaired *t*-test). In PAD hearts, ganglionic (22±0.9 ms, *P*=0.345, unpaired *t*-test), muscarinergic (24.4±0.7 ms, *P*=0.484, unpaired *t*-test) or β-blockade (25.2±0.5 ms, *P*=0.265, Mann–Whitney test) did not alter VRPs. However, cholinergic stimulation in PAD hearts (37.2±2.6 ms versus 23.4±0.9 ms, *P<*0.0001, unpaired *t*-test) prolonged refractory periods to similar values as in control hearts suggesting reversibility of PAD induced shortening of refractory periods. Electrophysiological parameters obtained *in vivo* and *ex vivo* are displayed in Supplementary Tables 1–4.

### PAD reduces dispersion of ventricular wave propagation

Next, we determined wave propagation velocity and dispersion in conduction direction by using multi-electrode epicardial activation mapping. Ventricular mapping during fixed pacing indicated that mean epicardial wave propagation velocity did not differ between PAD (0.77±0.05 m s^−1^) and control hearts (0.89±0.05 m s^−1^, *P*=0.09, unpaired *t*-test; Fig. 1e). Of interest, colour-coded reconstruction of isochrones in combination with arrows representing the direction of conduction showed that dispersion in conduction direction was significantly lower in PAD (0.52±0.07 radian) compared with control hearts (0.91±0.12 radian, *P*=0.026, Mann–Whitney test; Fig. 1f) indicating that cardiac cholinergic neurons impact the uniformity of conduction.

### Cholinergic PAD increases ventricular arrhythmia occurrence

**Figure 2:**
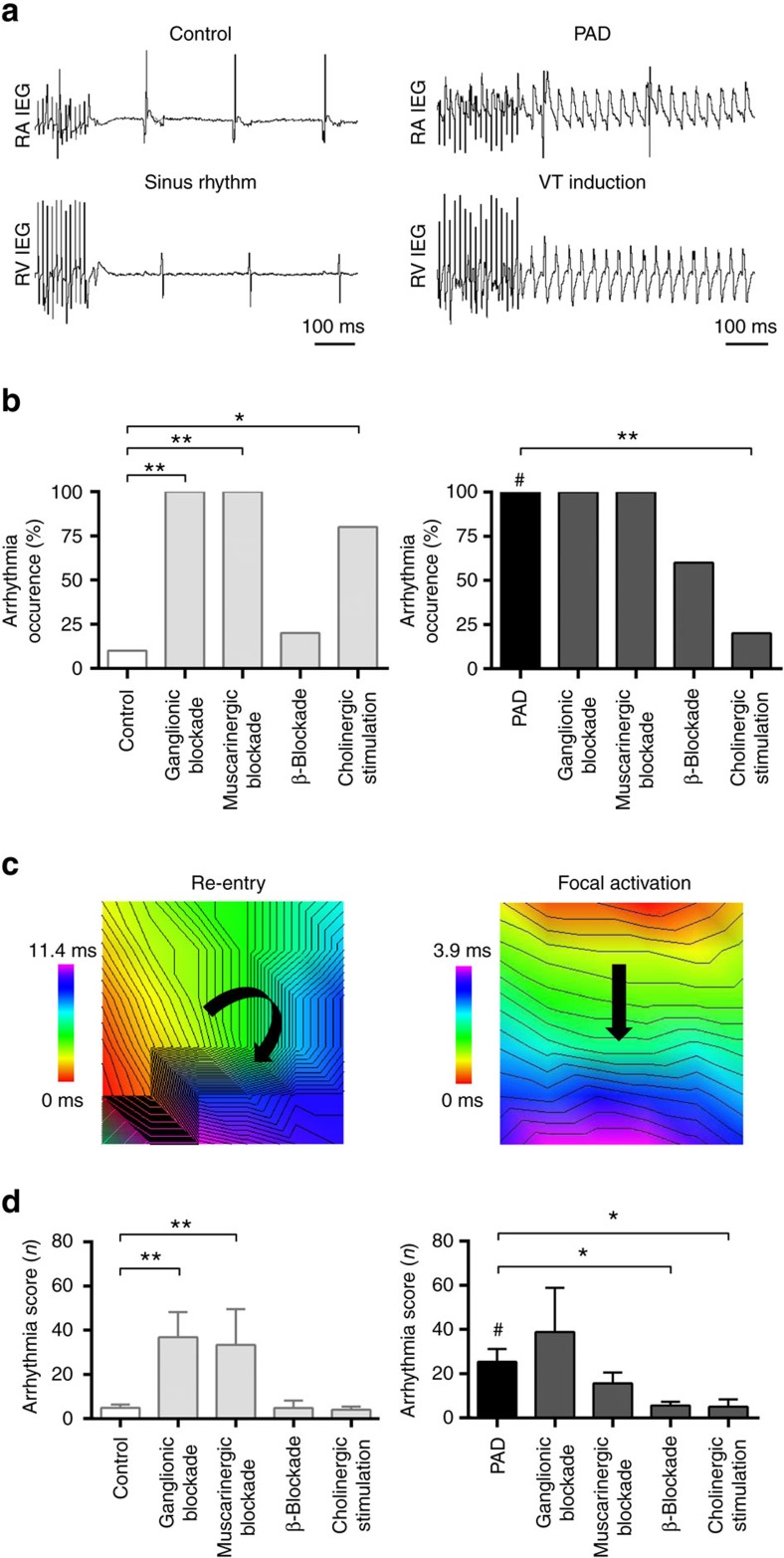
Disruption of atrial cholinergic control increases ventricular arrhythmogenesis. (**a**) Example of arrhythmia susceptibility testing using burst stimulation in the right ventricle (RV; cycle length 10 ms, duration 5 s.) without (left panel: control) or with (right panel: partial atrial denervation (PAD)) induction of a ventricular tachycardia (VT). Arrhythmias occurred more frequently in PAD hearts. RA, right atrium; IEG, intracardiac electrogram. (**b**) Susceptibility to ventricular arrhythmias increased after PAD (right panel, black bar, 100%, *n*=10) compared with control hearts (left panel, white bar, 10%, *n*=10; denoted by #, *P*<0.0001). In control hearts, ganglionic (hexamethonium, 5 × 10^−4^ M; *n*=5; *P*=0.002) or muscarinergic blockade (atropine, 1 × 10^−5^ M, *n*=5; *P*=0.002) increased arrhythmia susceptibility. β-Blockade (propranolol, 1 × 10^−6^ M; *n*=5) did not significantly influence arrhythmia inducibility in controls. Cholinergic stimulation (acetylcholine, 1 × 10^−5^ M, *n*=5; *P*=0.017) led to an increase in arrhythmia susceptibility in control hearts in line with previous findings^16^. In PAD hearts, ganglionic (*n*=5), muscarinergic (*n*=5) or β-blockade (*n*=5) did not significantly influence arrhythmia inducibility. Cholinergic stimulation reduced VT occurrence in PAD hearts (*n*=5; *P*=0.004). Fisher's exact test was used for all analyses in **b**. (**c**) Examples of ventricular arrhythmias with different underlying mechanisms (left: re-entry; right: focal). Red colour displays areas of earliest activation, purple of latest activation. (**d**) The arrhythmia score classifies the induced ventricular arrhythmias. After PAD, ventricular arrhythmias tended to be longer and more severe compared with controls (denoted by #; *n*=10; *P*=0.003; unpaired *t*-test), which is reflected by higher score values. In control hearts, ganglionic (*n*=5; *P*=0.002; unpaired *t*-test) or muscarinergic blockade (*n*=5; *P*=0.010; Mann–Whitney test) increased the arrhythmia score, while it was not affected by β-blockade or cholinergic stimulation. In PAD hearts, ganglionic (*n*=5) or muscarinergic blockade (*n*=5) did not alter the arrhythmia score. β-Blockade (*n*=5; *P*=0.011; Mann–Whitney test) or cholinergic stimulation (*n*=5; *P=*0.036; unpaired *t*-test) reduced it, indicating that PAD or pharmacological modulation of the cardiac autonomic tone impacts arrhythmia susceptibility. All the values shown are mean±s.e.m. *P<0.05, **P<0.01.

To investigate whether modulation of the intrinsic autonomic nervous system of the heart increases arrhythmia susceptibility, standardized programmed stimulation was performed (Fig. 2a–d). Compared with control hearts, we observed an increase in susceptibility to ventricular arrhythmias after PAD (10% versus 100%, *P*<0.0001, Fisher's exact test; Fig. 2b). In control hearts, ventricular arrhythmias occurred more frequently after ganglionic blockade (10% versus 100%, *P*=0.002, Fisher's exact test), muscarinergic blockade (10% versus 100%, *P*=0.002, Fisher's exact test) or cholinergic stimulation (10% versus 80%, *P*=0.017, Fisher's exact test) in line with previous findings^16^. β-Blockade (10% versus 20%, *P*=1.0, Fisher's exact test) did not alter arrhythmia susceptibility. In PAD hearts, ganglionic blockade (100% versus 100%, *P*=1.0, Fisher's exact test), muscarinergic blockade (100% versus 100%, *P*=1.0, Fisher's exact test) or β-blockade (100% versus 60%; *P*=0.095, Fisher's exact test) did not influence arrhythmia occurrence. Cholinergic stimulation reversed the increased susceptibility to arrhythmias in PAD hearts (20% versus 100%, *P*=0.004, Fisher's exact test).

Compared with controls (4.9±1.5), the ventricular arrhythmia score was higher after PAD (25.4±5.8, *P*=0.003, unpaired *t*-test; Fig. 2d). In control hearts, ganglionic blockade (36.8±11.4, *P*=0.002, unpaired *t*-test) and muscarinergic blockade (33.4±16.2, *P*=0.010, Mann–Whitney test) increased the arrhythmia score, while it was not altered during β-blockade (4.8±3.3, *P*=0.975, unpaired *t*-test) or cholinergic stimulation (4.0±1.4, *P*=0.702, unpaired *t*-test). In PAD hearts, ganglionic (38.8±20, *P*=0.745, Mann–Whitney test) or muscarinergic blockade (15.6±4.9, *P*=0.228, unpaired *t*-test) did not influence the arrhythmia score, while β-blockade (5.6±1.7, *P*=0.011, Mann–Whitney test) or cholinergic stimulation (5.0±3.4, *P*=0.036, unpaired *t*-test) reduced it. This suggests that PAD or pharmacological modulation of cardiac autonomic tone impacts arrhythmia susceptibility.

### Cardiac cholinergic neurons decrease ventricular cAMP levels

**Figure 3:**
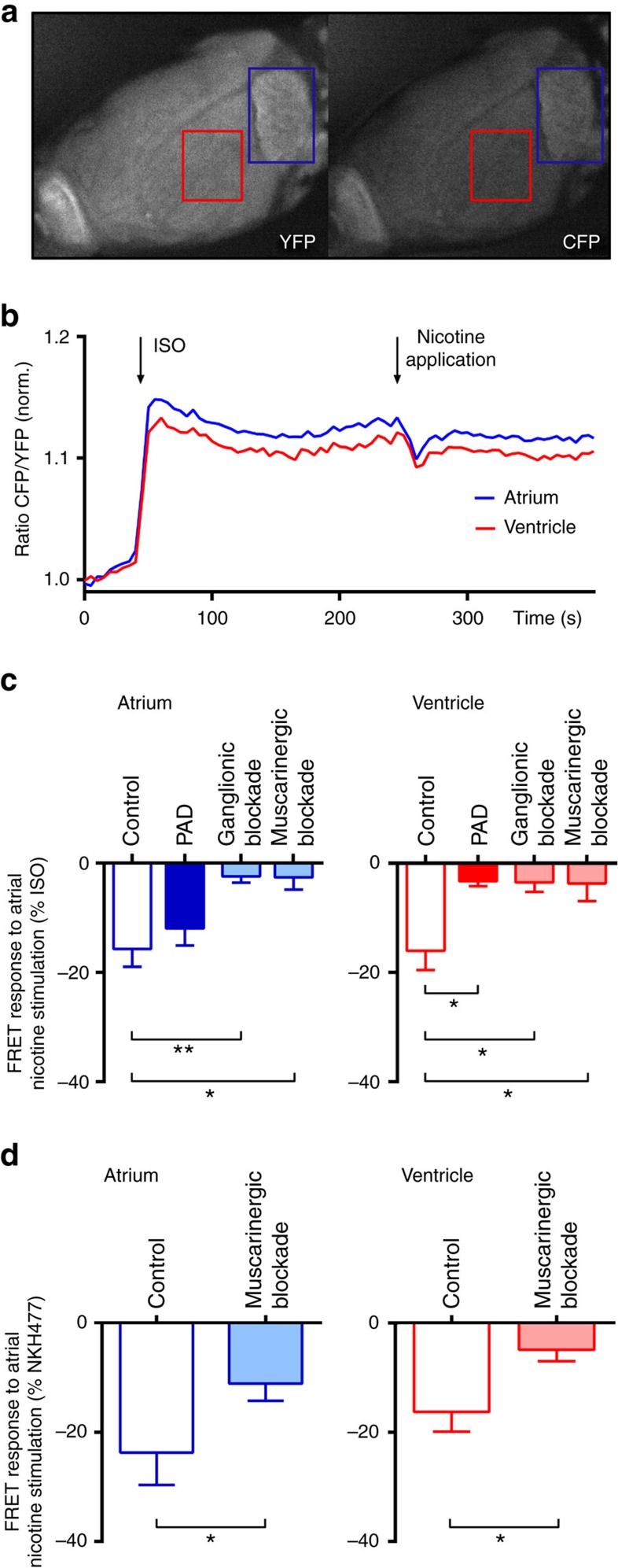
Atrial cholinergic modulation alters ventricular cAMP levels. (**a**) Cyclic adenosine monophosphate (cAMP) biosensor fluorescence in yellow (YFP) and cyan (CFP) channels during FRET measurements in a Langendorff-perfused heart. The red/blue squares at the ventricle/atrium indicate the areas of measurement. (**b**) Representative experiment depicting time-resolved cAMP dynamics (presented as normalized CFP/YFP ratio) during perfusion with isoproterenol (ISO, 1 × 10^−7^ M) and targeted atrial application of nicotine (6.2 × 10^−6^ M). cAMP levels in the left atrium and ventricle were decreased by nicotine. (**c**) Relative changes in cAMP levels (% of ISO response) in atria and ventricles during local atrial application of nicotine in control hearts without pharmacological intervention (*n*=8), after partial atrial denervation (PAD; *n*=5) and during ganglionic (hexamethonium, 5 × 10^−4^ M; *n*=5) or muscarinergic blockade (atropine, 1 × 10^−5^ M; *n*=5) are depicted. In the atrium, targeted atrial nicotine application reduced relative atrial cAMP levels in controls and PAD hearts. This effect was reduced after ganglionic blockade (*n*=5; *P*=0.009; unpaired *t*-test) or muscarinergic blockade (*n*=5; *P*=0.011; Mann–Whitney test). In the ventricle, relative changes in cAMP levels during targeted atrial application of nicotine was obvious in controls (*n*=8) but abolished in PAD hearts (*n*=5; *P*=0.018; unpaired *t*-test) and during ganglionic (*n*=5; *P*=0.023; unpaired *t*-test) or muscarinergic blockade (*n*=5; *P*=0.036; unpaired *t*-test), indicating an inhibition of parasympathetic activity. (**d**) After stimulation with the water-soluble forskolin analogue NKH477 (1 × 10^−5^ M; *n*=5), relative changes in cAMP levels (% of NKH477 response) during local atrial application of nicotine in control hearts were recorded for the atrium and ventricle. Muscarinergic blockade applied in the same hearts reduced cAMP levels in the atrium (*P*=0.019; paired *t*-test) and ventricle (*P*=0.039; paired *t*-test) of NKH477 stimulated hearts. All the values shown are mean±s.e.m. *P<0.05, **P<0.01.

To analyse effects of local atrial nicotinergic stimulation on atrial and left ventricular cAMP levels, we used a newly established whole-heart Förster Resonance Energy Transfer (FRET)-based imaging method (Fig. 3a,b). Topical application of nicotine (ganglionic stimulant) reduced isoproterenol (ISO)-stimulated atrial (−16±3.3%; Fig. 3c left panel) and ventricular (−16±3.5%; Fig. 3c right panel) cAMP levels in control hearts. The effect of nicotine on ventricular cAMP levels was almost completely abolished after PAD (−3.3±0.8%, *P*=0.018, unpaired *t*-test; Fig. 3c). Ganglionic (−3.5±1.7%, *P*=0.023, unpaired *t*-test) or muscarinergic blockade (−3.7±3.2%, *P*=0.036, unpaired *t*-test; Fig. 3c) also blocked the response. In the atrium, cAMP levels were as well reduced by local nicotine application (−16±3.3%). This effect was not altered by PAD (−12±3.1%, *P*=0.45, unpaired *t*-test). However, ganglionic (−2.4±1.1%, *P*=0.009, unpaired *t*-test) or muscarinergic blockade (−2.6±2.2%, *P*=0.011, Mann–Whitney test) reduced atrial cAMP levels after local nicotinergic stimulation. Absolute changes of cAMP levels after nicotinergic stimulation were 2.29 in the atrium and 0.89 in the ventricle. Similar effects have been observed after direct adenylyl cyclase activation using the soluble forskolin analogue NKH477 (Fig. 3d). Here, nicotine reduced NKH477 stimulated cAMP levels in the atrium (−24±5.9%) as well as in the ventricle (−16±3.6%), which were reduced after muscarinergic blockade (atrium: −11±3.1%, *P*=0.019, paired *t*-test; ventricle: −4.9±2.1%, *P*=0.039, paired *t*-test). Experiments in isolated cardiomyocytes confirmed changes in maximal cAMP levels (after ISO stimulation) due to cholinergic stimulation (−55±8.4%) or muscarinergic blockade (−3.8±6.7%, Supplementary Fig. 1). These results demonstrate that activation of atrial cholinergic neurons by nicotine modulates atrial and ventricular cAMP levels.

### Cholinergic nerves traverse from the atria to the ventricle

**Figure 4:**
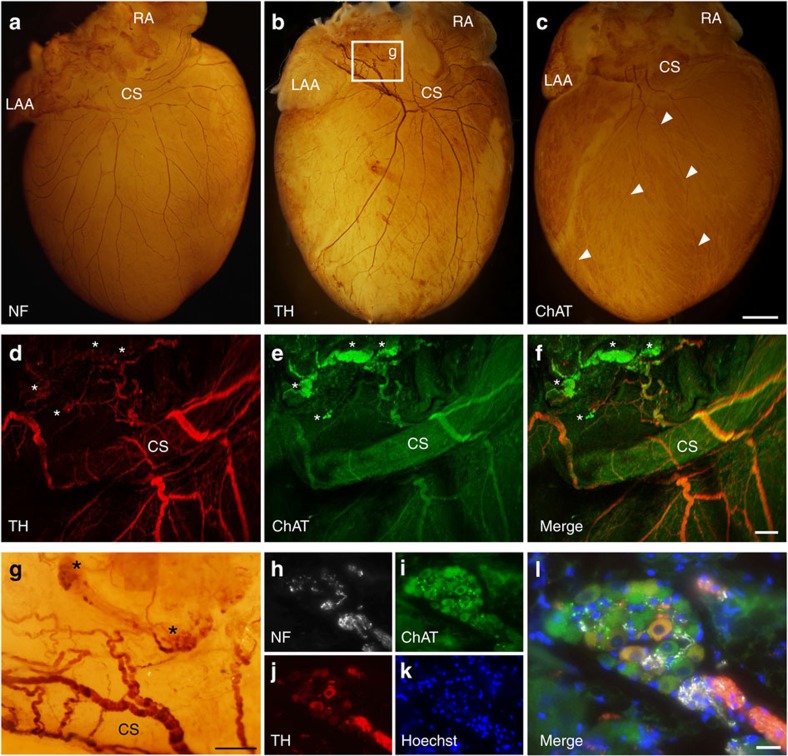
Interconnectivity of the atrial and ventricular intracardiac neural network. (**a**–**c**) Representative whole-mount stainings against neurofilament (NF; **a**), tyrosine hydroxylase (TH; **b**) and choline acetyltransferase (ChAT; **c**). Fibres traversed from the posterior left atrium along the pulmonary veins and the coronary sinus (CS) to the apex of the ventricle. Arrow heads mark the course of two ChAT-positive fibres. CS, coronary sinus; LAA, left atrial appendage; RA, right atrium. (**d**–**f**) Confocal imaging of TH (**d**) and ChAT (**e**) whole-mount co-staining revealed interwoven parasympathetic and sympathetic fibres originating from the parasympathetic ganglia (marked with asterisks). (**g**) Magnification from **b** depicting TH-positive fibres originating from atrial ganglia (marked with asterisks) with a dense network entwining the CS. (**h**–**l**) Immunohistochemical staining of atrial ganglia illustrated the predominance of ChAT-positive cells. Scale bars, 1 mm (**a**–**c**), 200 μm (**d**–**g**), 25 μm (**h**–**l**).

**Figure 5:**
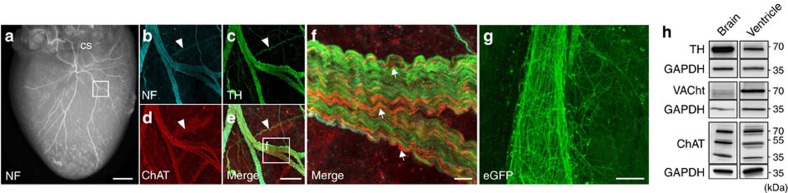
Sympathetic and parasympathetic ventricular innervation. (**a**) Neurofilament (NF) staining depicting the rich innervation of the ventricle. The square marks the location of the fibres shown in **b**–**f**. (**b**–**f**) Confocal imaging of ventricular NF (**b**), tyrosine hydroxylase (TH; **c**) and choline acetyltransferase (ChAT; **d**). The white arrow heads display a TH- but not ChAT-positive fibre. The greater magnification (**f**) emphasizes the tight entanglement of fibres, with the majority being TH-positive and a smaller amount of parallel running ChAT-positive fibres. Arrows depict ChAT-positive fibres. (**g**) Native enhanced green fluorescent protein (eGFP) fluorescence of ChAT^BAC^-eGFP mice demonstrated a delicate interconnectivity between parasympathetic ventricular fibres and cardiac myocytes. (**h**) Western blot analysis of ventricular tissue, using antibodies against TH, ChAT and VAChT revealed bands at the same molecular weight as in the brain (positive control), indicating the presence of a cholinergic system in the ventricles. Scale bars 1 mm (**a**), 100 μm (**b**–**e**), 10 μm (**f**), 25 μm (**g**).

**Figure 6:**
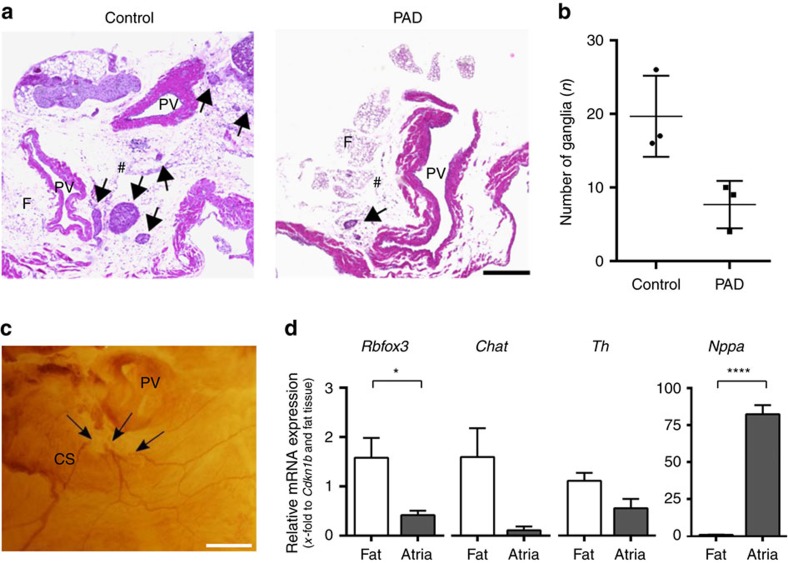
Quantification of cardiac ganglia after PAD. (**a**) Haematoxylin and eosin (H&E)-stained sections of control (left image) and partial atrial denervated (PAD) hearts (right image) are depicted. Cholinergic ganglia are marked with arrows. In PAD hearts, a great amount of epicardial fat and the ganglia located within have been removed. F, fat; PV, pulmonary vein; #, posterior atrial wall. Scale bar, 300 μm. (**b**) The total number of ganglia counted in the H&E-stained sections is reduced by 61% in PAD hearts compared with controls (*n*=3; *P*=0.100; Mann–Whitney test). (**c**) Whole-mount staining against choline acetyltransferase (ChAT) depicting disrupted cholinergic nerve fibres (arrows) after PAD. CS, coronary sinus. Scale bar, 500 μm. (**d**) Gene expression analysis of atrial myocardial tissue and epicardial fat via qRT–PCR (*n*=7) revealed significant differences in neuronal mRNA levels between the epicardial fat and cardiac atria. Compared with atrial tissue *Rbfox3* mRNA *(P*=0.011; paired *t*-test)*, Chat* mRNA (*P*=0.063; Wilcoxon signed-rank test) and *Th* mRNA *(P*=0.078; Wilcoxon signed-rank test) seemed to be slightly more prominent in the epicardial fat. Atrial *Nppa* expression (representing atrial natriuretic peptide) confirms correct fat preparation as it is only expressed within the atria *(n*=7; *P<*0.0001; paired *t*-test). All the values shown are mean±s.e.m. *P<0.05, ****P<0.0001.

To investigate the interconnectivity of the atrial and ventricular intracardiac autonomic nervous system, we examined the relation between cholinergic ganglia and sympathetic/parasympathetic neural fibres. Immunohistochemical staining of whole mount murine hearts revealed fibres originating from atrial ganglia, passing the coronary sinus towards ventricular myocardial tissue (Fig. 4a–l; Supplementary Tables 5 and 6). Choline acetyltransferase (ChAT)-positive ganglia were mainly located around the pulmonary veins. Both, sympathetic (tyrosine hydroxylase (TH)-positive) and parasympathetic (ChAT-positive) structures were located in close proximity to each other in the atria and along the coronary sinus. From there, axons traversed towards both ventricles via different routes (Fig. 5a–f). Up to the ventricular apex, TH-positive fibres were predominant and tightly intertwined with ChAT-positive fibres (Fig. 5f). Western blotting against ChAT and against the vesicular acetylcholine transporter (VAChT) confirmed the presence of a 70 kDa band (known to be the neuronal ChAT isoform^17^) in the ventricles that was also detectable in brain tissue (Fig. 5h; Supplementary Fig. 2), which indicates the presence of a cholinergic system in the ventricles. Native eGFP fluorescence of ChAT^BAC^-eGFP mice demonstrated a delicate network of parasympathetic fibres surrounding cardiac myocytes (Fig. 5g). PAD resulted in a 61% removal of ganglia which was revealed in haematoxylin and eosin-stained sections (7.7±1.9 versus 19.7±3.2 ganglia, *P*=0.1, Mann–Whitney test; Fig. 6) and whole-mount ChAT stainings of PAD hearts (6.2±0.3 ganglia). Quantitative real-time polymerase chain reaction (qRT-PCR) of the atria after PAD indicated a clear reduction of *Chat* mRNA (0.11±0.08-fold to *Cdkn1b* and fat tissue, *P*=0.063, Wilcoxon signed-rank test), as well as *Th* mRNA (0.55±0.2-fold to *Cdkn1b* and fat tissue, *P*=0.078, Wilcoxon signed-rank test) and *RBfox3* mRNA (also known as *NeuN*), which is expressed exclusively in neuronal nuclei^18^ (0.41±0.09-fold to *Cdkn1b* and fat tissue, *P*=0.011, paired *t*-test; Fig. 6d).

### PVC occurrence after AF ablation

**Figure 7:**
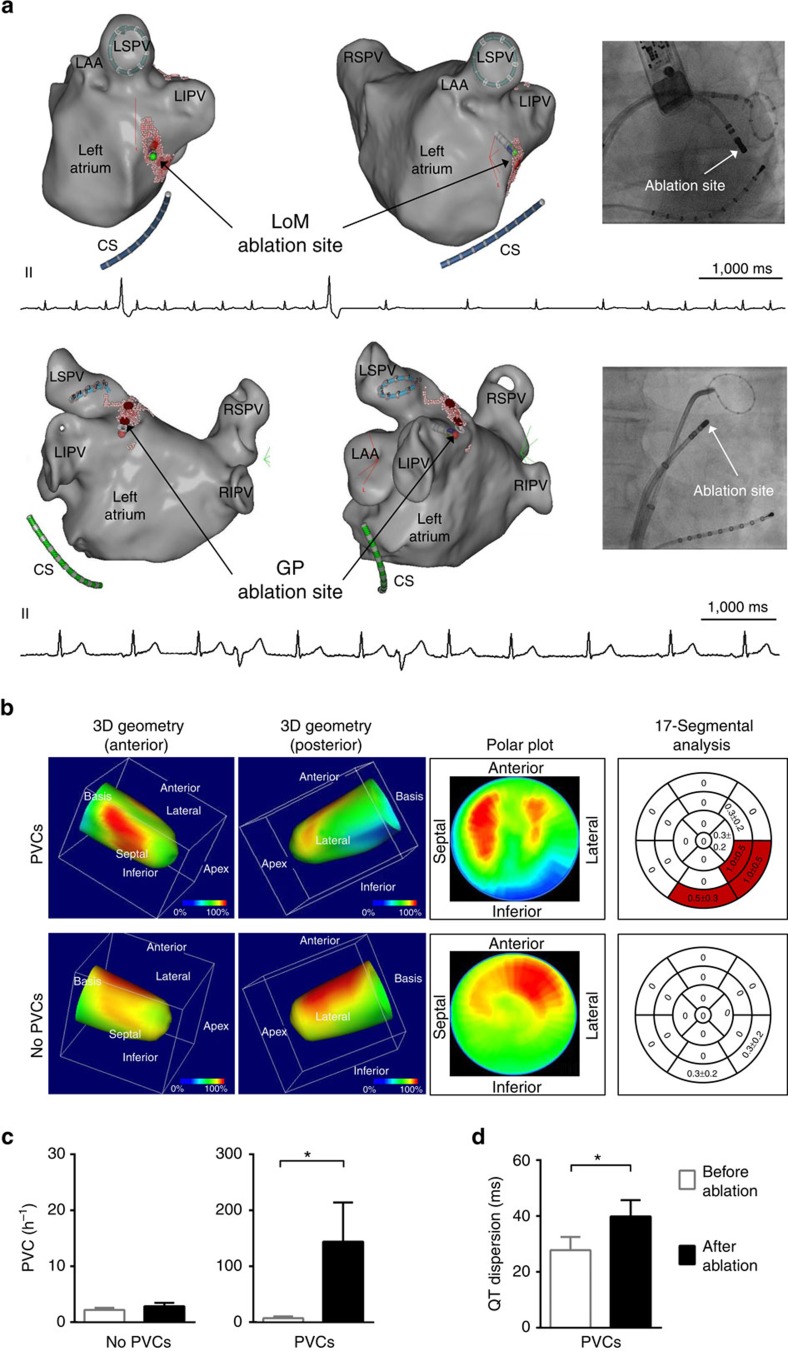
Atrial neuroablation affects ventricular electrophysiology. (**a**) Examples of three-dimensional (3D) guided ablation at the endocardial ligament of Marshall region (LoM; upper panel) and left superior ganglionated plexi (GP) region (lower panel) induces premature ventricular complexes (PVCs) during catheter ablation of AF. Red dots (VisiTag points) illustrate the sites of radiofrequency ablation. Left lateral (left) and anterolateral (right) views are shown. Note the PVCs occurring during ablation depicted in the ECG. A circular mapping catheter is located within the left superior pulmonary vein (LSPV; lower panel). The fluoroscopy image at the right shows the position of the ablation catheter and the circular mapping catheter positioned within the left inferior pulmonary vein (LIPV; upper panel) demonstrating the close proximity between this vein, the coronary sinus and the ablation site. CS, coronary sinus; LA, left atrium; LAA, left atrial appendage; right inferior pulmonary vein, RIPV; right superior pulmonary vein, RSPV. Scale bar, each 1,000 ms. (**b**) I-123-MIBG SPECT imaging revealed a reduced sympathetic innervation at the inferior and inferolateral parts of the left ventricle after catheter ablation of AF in patients with an increased PVC burden or symptomatic palpitations after AF ablation (*n*=4). In those without an increase in PVCs after AF ablation (*n*=4), regional left ventricular sympathetic innervation was less impaired. The right panel displays mean summed defect score values, whereas red parts depict areas of reduced uptake (mean summed defect score for one segment ≥0.5). (**c**) The majority of patients had no increase of PVCs per hour (No PVCs, *n*=105). A subset of patients (PVCs, *n*=6; *P*=0.031; Wilcoxon signed-rank test) reported palpitations or shortness of breath after catheter ablation of AF concomitant with an increase in PVCs per hour, irrespective of AF recurrence. (**d**) QT dispersion after catheter ablation was increased in patients with an elevated PVC burden (*n*=6; *P*=0.011; paired *t*-test). All the values shown are mean±s.e.m. *P<0.05.

To investigate whether modulation of the intrinsic cardiac autonomic nervous system at the atrial level impacts ventricular electrophysiology in humans, we studied 111 consecutive patients (60±1 years, 60% male) undergoing catheter ablation of paroxysmal AF (Supplementary Table 7). During ablation at anatomical sites of cardiac ganglia in the left atrium (Ligament of Marshall region, *n*=5; left superior ganglionated plexus region, *n*=1; Fig. 7a) premature ventricular complexes (PVCs) occurred in six patients with concomitant vagal responses in five patients.

In 20% of all patients, atrial arrhythmias were detected during follow-up after a blanking period of 3 months, irrespective of PVC incidence. During follow-up, in six patients a PVC increase was documented irrespective of AF recurrence (before catheter ablation: 7±3.3 PVC h^−1^; after catheter ablation: 144±70 PVC h^−1^, *P*=0.031, Wilcoxon signed-rank test; Fig. 7c). Meanwhile, QT dispersion (28±5 ms versus 40±6 ms, *P*=0.011, paired *t*-test; Fig. 7d) as a marker of repolarization heterogeneity increased after catheter ablation. In one of these patients, a non-sustained ventricular tachycardia (VT) was detected during follow-up. In age- and gender-matched controls (*n*=6) without a significant increase in PVCs per hour after AF ablation (1.4±1.3 PVC h^−1^ versus 2.4±2.0 PVC h^−1^, *P*=0.5, Wilcoxon signed-rank test), the ventricular repolarization heterogeneity did not change (QT dispersion: 22±3 ms versus 24±3 ms, *P*=0.222, paired *t*-test).

### AF ablation reduces parasympathetic and sympathetic activity

The mean heart rate increased from 61±1 b.p.m. before to 69±1 b.p.m. (*P*<0.0001; paired *t*-test) 48 h after AF ablation in all patients with sinus rhythm, indicating a reduction of cholinergic control. Heart rate variability (HRV) and HRV measurement during deep breathing (HRV-DB) indicated a reduced tonic and phasic parasympathetic activity after catheter ablation of AF (Supplementary Table 8).

In patients with an increase in PVCs after AF ablation, the heart rate increased ≥10 b.p.m. in four out of six patients (59±4.4 b.p.m. versus 64±2.5 b.p.m., *P*=0.34, paired *t*-test) suggesting parasympathetic denervation, while it did not change in controls (66±5.4 b.p.m. versus 67±3.3 b.p.m., *P*=0.81, Wilcoxon signed-rank test).

Cardiac I-123-meta-iodo-benzylguanidine (MIBG) single-photon emission computed tomography (SPECT) imaging demonstrated a heart-to-mediastinum ratio of 1.61±0.05 in patients with new onset of PVCs (*n*=4) and 1.56±0.12 (*P*=0.83; Mann–Whitney test) in patients without PVCs after AF ablation (*n*=4). Semiquantitative analysis of sympathetic denervation revealed a summed defect score of 3.0±1.8 with regional defects of sympathetic innervation at the inferior and inferolateral left ventricular wall in patients with PVCs after AF ablation (Fig. 7b). Sympathetic innervation was less impaired in patients without PVCs after AF ablation (summed defect score 0.5±0.5, *P*=0.43; Mann–Whitney test) indicating a relation between the modulation of atrial cholinergic neurons, concomitant sympathetic denervation and the occurrence of ventricular arrhythmias.

## Discussion

The major findings of the present study are as follows: (i) morphological and functional evidence support that cardiac cholinergic neurons regulate ventricular electrophysiology; (ii) modulation of the atrial neural network changes left ventricular cAMP levels; (iii) accidental partial atrial denervation during catheter ablation of AF is characterized by both reduced parasympathetic activity and disturbed sympathetic ventricular control. This can translate into increased susceptibility to ventricular arrhythmia after catheter ablation of AF.

It is well known that catheter ablation of AF inevitably modulates the intracardiac neural network as supported by morphological and functional studies^2^. The most obvious clinical parameter is an increased heart rate, which is often used as a surrogate parameter for withdrawal of sinus nodal or global cardiac parasympathetic activity following catheter ablation of AF^19^,^20^. Our indirect measurements of neural cardiac control including heart rate, HRV at rest and HRV-DB, support a decrease in both tonic and phasic parasympathetic activity after catheter ablation of AF in our patient cohort. The former is in line with several previous reports having found an impairment of HRV following AF ablation^21^. Although still controversial, observational studies^22^ and one randomized controlled trial^5^ support that atrial denervation (accidentally or targeted) can reduce AF recurrence. Importantly, PVCs and VTs have been reported following AF catheter ablation in patients without any evidence of structural heart disease or inherited ion channel diseases^23^,^24^,^25^. Our results support reports having speculated that these arrhythmias might reflect autonomic influences^26^.

Atrial and ventricular neural structures are tightly interconnected^27^,^28^. In addition, some studies support textbook knowledge stating that beyond sensory afferents, ventricular electrophysiology is primarily controlled by postganglionic sympathetic efferent axons^6^,^29^. Although controversial, ventricular innervation by parasympathetic fibres^7^,^8^ and changes in ventricular repolarization following cervical vagal nerve stimulation have been described^11^,^14^,^30^. This influence of extracardiac vagal stimulation on ventricular electrophysiology is relayed via different intracardiac ganglia^15^,^30^ but it is not fully understood whether this effect is mediated via central or peripheral neural pathways. Here, we present morphological and functional evidence demonstrating that cardiac cholinergic neurons modulate ventricular electrophysiology.

Stimulation of post-ganglionic neurons decreased ISO- and Forskolin-stimulated cAMP levels in the ventricle, which was blocked by PAD, the ganglion nicotinic antagonist hexamethonium, or the muscarinic antagonist atropine, indicating that cholinergic neurons modulate ventricular function. This is also supported by PAD-induced shortening of the VRP and immunohistochemical demonstration of intertwined TH- and ChAT-positive fibres in the ventricle. However, one should keep in mind that the behaviour of these neurons may be completely different from *in vivo* conditions, as central inputs have been disrupted.

It also needs to be taken into account, that isolated cardiomyocytes themselves have been shown to synthesize acetylcholine^31^,^32^. Although it is not clear whether and how this non-neuronal acetylcholine acutely modulates ventricular electrophysiology, it might have influenced our results at least in part. However, since acetylcholine was only detectable after the addition of acetylcholine esterase inhibitors in isolated myocytes^31^,^32^, it seems unlikely that non-neuronal acetylcholine explains the functional effects observed in our study.

It is well known, that besides parasympathetic also sympathetic activity can influence ventricular electrophysiology. Our experimental and clinical data suggest that both the reduction of parasympathetic activity as well as the concomitant heterogeneity in ventricular sympathetic predominance are accountable for the increase in arrhythmia susceptibility. Although the importance of sole decreased parasympathetic activity in structurally healthy hearts is less well defined, it is widely accepted that disturbed sympathetic/parasympathetic interactions are proarrhythmic^33^,^34^. It has been described that muscarinic receptors on adrenergic nerve terminals attenuate norepinephrine release^35^, leading to parasympathetically mediated inhibitory effects on cardiac sympathetic activity. This indirect parasympathetic ventricular control by opposing the effects of heightened adrenergic tone might influence ventricular electrophysiology in Langendorff-perfused hearts. However, β-blockade^36^ did not significantly influence arrhythmogenesis in PAD hearts in our studies. PAD as well as ganglionic or muscarinergic blockade led to an increased susceptibility to ventricular arrhythmias. This indicates that intracardiac neurons continue to fire action potentials in an *ex vivo* setup and are able to communicate with each other^36^,^37^.

In addition, FRET imaging confirmed the influence of atrial neurons via a reduction of ventricular cAMP levels during local application of nicotine, although these experiments were performed under conditions of sympathetic stimulation or direct cAMP activation. This influence on ventricular control was abolished by muscarinergic or ganglionic blockade, while cholinergic stimulation of PAD hearts prevented VT inducibility. However, although some major patterns are similar in mouse and man, extrapolating murine data to human studies may be difficult as cardiac neuroanatomy is known to exhibit interspecies variability^38^.

In conclusion, modulation of atrial cholinergic neurons can acutely influence ventricular electrophysiology. Reduced parasympathetic activity and concomitant spatial heterogeneity of dominant sympathetic activity can translate into increased ventricular arrhythmogenesis in some patients after catheter ablation of AF.

## Methods

### Animals

The study protocol was approved by the local authorities of the State of Hamburg, the University of Hamburg Animal Care and Use Committees and conforms to the Guide for the Care and Use of Laboratory Animals, Eighth Edition, National Academy Press, updated by the US National Research Council Committee in 2011.

### Electrophysiological studies

We examined male wild-type C57/BL6 mice (10 to 16 weeks of age; Stock number Jackson Laboratories: 000664) electrophysiologically by a single catheter technique^39^. To obtain baseline cardiac electrophysiological, electrocardiographic (ECG) and echocardiographic parameters, *in vivo* studies (*n*=10) were performed (Supplementary Tables 1–4)^39^. The mice were anaesthetized with 2.4 Vol.% isoflurane and positioned on a temperature-controlled platform to maintain body core temperature at 36 °C. Surface resting ECG was obtained and then analysed offline by using Powerlab 8/30 & Labchart (ADInstruments, Dunedin, New Zealand). For echocardiography, the parasternal long-axis view, short axis view and two-dimensional-guided M-mode images were obtained at the level of the papillary muscles for the measurement of wall thickness and chamber dimensions. Electrophysiological parameters like the sinus node recovery time (maximum return cycle length after 10 s fixed-rate pacing), Wenckebach periodicity (longest cycle length with loss of 1:1 atrioventricular-nodal conduction), atrioventricular-nodal refractory period (longest extrastimulus cycle length with loss of atrioventricular-nodal conduction), atrial and ventricular refractory periods (longest extrastimulus cycle length with absent atrial or ventricular response) were determined by using fixed-rate or extrastimulus pacing. In *ex vivo* Langendorff studies, the epicardial atrial fat pads, in which atrial ganglia are mainly located, were either removed by careful dissection^40^ (PAD) or left intact (control). For electrophysiological studies in a Langendorff setup, hearts were submerged in ice-cold modified Krebs-Henseleit solution (mM: NaCl 119, NaHCO_3_ 25, KCL 4.6, KH_2_PO_4_ 1.2, MgSO_4_ 1.1, CaCl_2_ 2.5, glucose 8.3 and Na-pyruvate 2; pH 7.4, 95% O_2_/5% CO_2_) and dissected carefully. The aorta was cannulated, quickly attached to the perfusion system (Hugo Sachs Elektronik/Harvard Aparatus, Germany) and retrogradely perfused at constant pressure (80 mm Hg; ref. 41). The heart was surrounded by a fully closed chamber, ensuring a precise temperature control. A 2F octapolar electrophysiology catheter with 0.5 mm electrode spacing (CIB'ER Mouse, NuMed Inc., Hopkinton, NY, USA) was inserted into the right atrium and right ventricle for recording of intracardiac electrograms via an ECG amplifier (F104, ADInstruments). A second 2F octapolar catheter located epicardially at the left ventricle stimulated the heart with a cycle length of 100 ms for the initial 20 min equilibration period. Hearts that did not regain a pink colouration or a spontaneous heart beat after equilibration were excluded. Perfusion pressure, aortic flow and heart rate were continuously recorded using a digital data acquisition system and corresponding software (Powerlab 8/30 & Labchart, ADInstruments). Programmed stimulation was applied via the distal or proximal electrodes of the catheter using a designated digital stimulus generator (STG4002, Multi Channel Systems, Reutlingen, Germany) at twice the atrial or ventricular pacing threshold to determine standard electrophysiological parameters^42^. In line with the Lambeth Conventions^43^, programmed extrastimulation techniques (baseline cycle lengths 100 ms and 120 ms) with up to three extrastimuli and burst pacing with a 2 ms or 10 ms stepwise reduction were used to detect arrhythmia susceptibility^44^,^45^,^46^. VT was defined as ≥4 consecutive premature ventricular complexes^43^. Ventricular arrhythmias have been classified by an established scoring system^47^.

In addition to PAD (*n*=10) and control hearts (*n*=10), further experiments (each *n*=5) were performed with the following drugs added to the perfusion solution before starting the experiment: hexamethonium (ganglionic blockade, 5 × 10^−4^ M, Sigma-Aldrich, St. Louis, MO, USA; *n*=5), atropine (muscarinergic blockade,1 × 10^−6^ M, Sigma-Aldrich; *n*=5), propranolol^48^ (β-receptor blockade, 1 × 10^−6^ M, mibe GmbH, Sandersdorf-Brehna, Germany; *n*=5), and acetylcholine (cholinergic stimulation, 1 × 10^−5^ M, Sigma-Aldrich; *n*=5). Pharmacological interventions were performed in additional experiments and only one drug was tested per heart (control or PAD) to keep examination time similar and to guarantee comparability in all performed experiments^49^.

### Epicardial mapping of Langendorff-perfused hearts

Myocardial wave propagation characteristics were determined by epicardial activation mapping with two 32-electrode arrays (EcoFlexMEA36, Multi Channel Systems, Reutlingen, Germany; inter-electrode distance: 300 μm; 1.8 × 1.8 mm) positioned at both ventricles during epicardial pacing (cycle length 100 ms) and induced ventricular arrhythmias. Unipolar electrograms were recorded (ME128-FAI-MPA-System, Multi Channel Systems, Reutlingen, Germany) with a sampling rate of 25 kHz. Data were bandpass filtered (50 Hz), digitized with 12 bit and a signal range of 20 mV. Wave propagation velocity and dispersion in conduction direction were determined^50^.

### FRET-based cAMP imaging in whole hearts and cardiomyocytes

Förster resonance energy transfer (FRET)-based measurements were performed in Langendorff-perfused hearts harvested from CAG-Epac1-camps transgenic mice^51^. After measurement of baseline activity, the hearts were perfused with isoproterenol (ISO, 1 × 10^−7^ M) or the NKH477 (1 × 10^−5^ M, both from Sigma-Aldrich) and subsequently stimulated with nicotine (40 μl of 6.2 × 10^−6^ M, Sigma-Aldrich), which was pipetted on the upper part of the left atrium^52^. These experiments were performed in control (*n*=8) or PAD hearts (*n*=5) and with perfusion of atropine (muscarinergic blockade, 1 × 10^−6^ M, Sigma-Aldrich; *n*=5) or hexamethonium (ganglionic blockade, 5 × 10^−4^ M, Sigma-Aldrich; *n*=5). In hearts that were prestimulated with NKH477, atropine (muscarinergic blockade, 1 × 10^−6^ M, Sigma-Aldrich) was added directly to the perfusion buffer before performing an additional nicotine stimulation. For single cell FRET measurements, adult murine cardiomyocytes were freshly isolated via retrograde cardiac perfusion with an enzyme solution containing 1.25 mg liberase DH (Roche Diagnostics Deutschland GmbH, Mannheim, Germany) and 300 μl trypsin (2.5%, Thermo Fisher Scientific, Waltham, MA, USA). After digestion, the calcium level was adapted and the cells were plated onto glass-bottomed, laminin (Sigma-Aldrich) coated dishes^53^. To monitor FRET, we used self-built imaging systems around Leica M165FC (Leica Microsystems GmbH, Wetzlar, Germany) stereomicroscope (for Langendorff experiments) or Leica DMI3000B (Leica Microsystems GmbH) inverted fluorescence microscope (for single-cell recordings). cAMP sensor was excited with 440 nm LED (pE-100, CoolLED, Andover, UK). Emission light was split into donor and acceptor channels using the DV2 DualView equipped with the 565dcxr dichroic mirror and D480/30 and D535/40 emission filters (Photometrics, Tucson, AZ, USA). Images were taken using optiMOS camera (Photometrics, Tucson, AZ, USA) with MicroManager 1.4 open source imaging software and analysed by Image J (NIH, USA). Raw data were corrected offline for the bleedthrough factor of the donor into the acceptor channel^54^.

### Histology and immunohistochemistry

Whole-mount immunostainings were performed in intact murine hearts to examine the interconnectivity of atrial and ventricular neural structures within the intracardiac neural network and cholinergic ventricular innervation^55^. Neurofilament staining (chicken anti NF-H, 1:3,000; EMD Millipore) was used to characterize the intracardiac neural network traversing along the pulmonary veins, the posterior left atrium and both ventricles. We used fluorescent and chromogenic labelling for TH and ChAT (goat α ChAT, 1:50; EMD Millipore; rabbit α TH, 1:1,000; EMD Millipore) to characterize adrenergic and cholinergic structures^35^,^55^,^56^. Male ChAT^BAC^-eGFP transgenic mice were purchased from Jackson Laboratories (Bar Harbor, ME, USA; stock number 007902; ref. 57). For imaging of native eGFP fluorescence, hearts were extracted and perfused with ice-cold PBS.

### Immunohistochemistry—whole-mount staining

After Langendorff perfusion, mouse hearts were fixed in formalin (Sigma-Aldrich) for 24 h at 4 °C and stored in phosphate-buffered saline (PBS, Biochrom GmbH, Germany) until the staining was performed. Hearts were bleached in Dent's bleach (4:1:1 MeOH: H_2_O_2_: DMSO, Merck KGA, Darmstadt, Germany) for 1 week at 4 °C and subsequently rehydrated to PBS in a series of descending MeOH in PBS (100, 75, 50, 25%, 1 h each)^58^. The following incubations were performed in 24-well-plate format with gentle agitation at 4 °C. The hearts were permeabilized in 1% Triton X-100/PBS (PBS-T, Sigma-Aldrich) for 3 × 1 h at room temperature before blocking overnight in blocking buffer (5% BSA/PBS-T (Biomol, Hamburg, Germany)+0.2% sodium azide). Antibodies were diluted as follows: goat α ChAT (1:50; EMD Millipore), rabbit α TH (1:1,000; EMD Millipore), chicken α neurofilament (1:3,000; EMD Millipore); for fluorescent labelling: donkey α rabbit IgG Alexa 488 (1:500; Thermo Fisher Scientific), donkey α goat IgG Alexa 568 (all 1:500; Thermo Fisher Scientific) and donkey α chicken IgY Alexa 647 (all 1:500; Thermo Fisher Scientific); for chromogenic labelling: biotin-conjugated donkey α rabbit igG (1:200; EMD Millipore), biotin-conjugated donkey α goat igG (1:200; R&D Systems), biotin-conjugated goat α chicken igY (1:200; R&D Systems); see also Supplementary Tables 5 and 6.

Specimens were incubated in primary antibodies diluted in blocking buffer for 1 week. Afterwards, the hearts were washed 3 × 15 min in PBS-T before secondary antibody incubation in blocking buffer for 4 days. Subsequently, the hearts were washed 3 × 15 min in PBS-T and stored in Vectashield mounting medium (#H-1000, Vector Laboratories, Burlingame, CA, USA) for fluorescent staining or incubated in Vectastain ABC kit (#PK-4000, Vector Laboratories) according to the manufacturer's instructions for 3 h at room temperature. Subsequently, the hearts were pre-incubated 1 h in Steady DAB/Plus (#ab103723, Abcam plc, Cambridge, UK) without DAB, before developing under visual control in Steady DAB according to the manufacturer's instructions. The specimens were stored in double-distilled H_2_O.

### Histology—paraffin sections

Mouse hearts were prepared and fixed as described above and subsequently dehydrated and embedded in paraffin. Four micrometre-thick sections were cut and deparaffinized using HistoClear (#HS2002, DiaTec, Bamberg, Germany) and ethanol. No antigen retrieval was performed. The slides were permeabilized for 10 min in 0.2% Triton X-100/Tris-buffered saline (TBS), followed by 3 × 5 min washes in TBS. Blocking was performed with 3% BSA/TBS for 1 h at room temperature. Antibodies were diluted as follows: goat α ChAT (1:50; EMD Millipore), rabbit α TH (1:500; EMD Millipore), chicken α neurofilament (1:1,000; EMD Millipore), donkey α rabbit IgG Alexa 488 (1:500; Thermo Fisher Scientific), donkey α goat IgG Alexa 568 (1:500; Thermo Fisher Scientific) and donkey α chicken IgY Alexa 647 (1:500; Thermo Fisher Scientific; see also Supplementary Tables 5-6) in 1% BSA/TBS. Incubation was performed overnight at 4 °C (primary antibody) or 2 h at room temperature (secondary antibodies) with 3 × 5 min washes TBS in between. 1 μg ml^−1^ bisBenzimide H33342 trihydrochloride (Hoechst, #B2261, Sigma-Aldrich) was added to the secondary antibody solution. The slides were mounted in Vectashield HardSet mounting medium (#VEC-H-1400, Vector Laboratories).

For quantification of cardiac ganglion removal, control and PAD hearts were cut in 5 μm thick sections, haematoxylin and eosin stained, partitioned in four regions and the number of ganglia was counted microscopically in a standardized way by two independent scientists (K.S. and N.B.)^59^.

### Microscopy

Confocal images of fluorescent whole mount and paraffin section stainings were taken with either a Leica TCS SP5 (Leica Microsystems GmbH) using × 10 NA=0.3 HCPL Fluotar, × 20 NA=0.7, HC PL Apo CS Imm/Corr oil and × 40 NA=1.3 HCX PL APO CS objectives or a 2-photon BX61WI upright microscope (Olympus Cooperation, Tokyo, Japan) using a plan-Neofluar × 5 NA=0.15 objective. Three-dimensional multicolour images were collected over the full range of the signal with sequential channel acquisition of each stack for whole mounts. A maximum projection image was created using the Leica LASAF software. Images of chromogenic stainings and overview of whole fluorescent hearts were photographed using an Olympus SZX16 wide zoom stereo microscope (Olympus Cooperation). Three different focal planes were collected for chromogenic stainings and stacked using the Helicon Focus Software (HeliconSoft Ltd.). If necessary, brightness and contrast was adapted using GNU Image manipulation programme (GIMP). For quantification of ganglia in whole-mount ChAT-stained hearts, ganglia were counted manually on the SZX16 microscope.

### Immunoblotting

Expression of sympathetic and parasympathetic markers was analysed in cardiac ventricular tissue via immunoblotting. Brain tissue served as positive control. Tissues were lysed in RIPA buffer (50 mM Tris base pH 8.0, 150 mM sodium chloride, 0.5% sodium deoxycholate, 0.1% sodium dodecyl sulfate (SDS), 1% Triton X-100, 1 mM dithiothreitol, plus proteaseinhibitors (Complete Mini, Roche Diagnostics Deutschland GmbH) and phosphataseinhibitors (phosSTOP, Roche Diagnostics Deutschland GmbH) using a glass-teflon potter and centrifuged for 30 min at 15.000*g*, 4 °C (ref. 60). Protein concentrations in the homogenates were quantified using the Pierce BCA Protein Assay Kit (Thermo Fisher Scientific) according to the manufacturer's instructions. The separation of 25 μg total protein was performed under denaturing conditions by sodium dodecyl sulfate-polyacrylamide gels (SDS–PAGE) using precast gels (4–20%, Bio-Rad, Hercules, CA, USA) and transferred to nitrocellulose membranes via wet blotting. The membranes were blocked in 5% skim milk powder in TBS. For goat-derived antibodies, blocking was performed in 5% BSA/TBS (receptor grade, Serva, Heidelberg, Germany). The membranes were incubated overnight at 4 °C with primary antibodies as follows: goat α ChAT (1:500, EMD Millipore), rabbit α TH (1:1,000; EMD Millipore), goat α VAChT (1:500, EMD Millipore); see also Supplementary Table 5. After three washing steps with TBS+0.5% Tween, secondary antibody incubation followed for one hour at room temperature: POX-conjugated horse α goat igG (1:10,000; Vector Laboratories) and POX-conjugated goat α rabbit igG (1:10,000; Vector Laboratories). Incubation with HRP-conjugated rabbit α GAPDH 14C10 (1:2,000; Cell Signaling) for 1 h was performed to detect GAPDH as a loading control. The Fusion Solo S gel documentation system (VRW International, Radnor, PA, USA) was used to detect reactive protein bands with enhanced chemiluminescence.

### RNA isolation and qRT–PCR

RNA was isolated using Trizol (Thermo Fisher Scientific) and further purified using the RNeasy Mini kit (Qiagen, Hilden, Germany) according to the manufacturer's instructions. Total RNA was reverse transcribed using the High-Capacity cDNA Reverse Transcription Kit (Thermo Fisher Scientific). qRT-PCR was performed on a 7900 TaqMan system (Applied Biosystems, Foster City, CA, USA) using TaqMan gene expression mastermix and gene expression assays (*Rbfox3* Mm01248771_m1; *Chat* Mm01221880_m1; *Th* Mm00447557_m1; Thermo Fisher Scientific). *Nppa* (mNPPA_Mm01255748_g1) was used as a control for atrial tissue preparation, 10-40 ng of cDNA were used as reaction template; experiments were performed in duplicates. Cycling conditions were as follows: 50 °C for 2 min; 95 °C for 10 min (one cycle), 95 °C for 15 s and 60 °C for 1 min (40 cycles). Relative gene expression was calculated using the 2^−ΔΔCt^ method, normalizing to *Cdkn1b* (Mm00438167_g1) and fat tissue^61^. *Cdkn1b* is not differentially expressed in fat and atrial tissue.

### Study protocol

All patients gave written informed consent. Patient studies were approved by the local Ethics Committee (No.: WF-01/16) and in accordance with the Declaration of Helsinki. The clinical trial registration can be found with the following URL (https://clinicaltrials.gov) under the unique identifier NCT02699255. The burden of ventricular arrhythmias was investigated in an observational study including 111 consecutive patients with paroxysmal AF undergoing catheter ablation. All patients underwent catheter ablation of AF using radiofrequency or cryoablation by an experienced electrophysiologist (>1,000 left atrial catheter ablation procedures each) and were continuously monitored for 48 h after the procedure. Ganglia were not identified by high-frequency stimulation-evoked bradycardia. Vagal responses during radiofrequency application were defined as a decrease of heart rate by ≥20% (ref. 62). Heart rate analysis was performed only in patients, which were in sinus rhythm before and after the procedure (*n*=93). The patients were asked to visit an outpatient clinic 3 and 6 months after the catheter ablation; 12-lead ECG or 24 h Holter monitoring was performed on the patients at every visit and whenever patients complained about symptoms. Antiarrhythmic drug therapy was discontinued after catheter ablation of AF. All patients continued oral anticoagulation for a minimum of 3 months. AF recurrence was defined as any episode of atrial tachycardia (AF, atrial flutter, atrial tachycardia) of at least 30 s in duration. In patients with cardiac implantable electrical devices, interrogation for arrhythmia detection was additionally performed. Patients with previous documentation of >30 PVCs h^−1^ (ref. 63), salvos or VTs, myocardial infarction, open heart surgery or percutaneous transluminal coronary angioplasty within less than 2 months before first AF ablation were excluded. For asymptomatic (>30 PVCs h^−1^) or symptomatic (onset of palpitations or shortness of breath) patients, experiencing a PVC increase after catheter ablation of AF, the QT dispersion was analysed as a marker of ventricular repolarization heterogeneity before and after catheter ablation by two independent investigators (C.J. and T.S.)^64^. Patients with an increase in PVCs (*n*=6) after AF ablation and an age- and sex-matched control group (*n*=6) were analysed^65^.

### Characterization of cardiac neural control

Parameters of cardiac neural control were investigated in a subset of 10 patients (64±10 years) before and after catheter ablation of AF. For indirect assessment of tonic parasympathetic activity, HRV analysis was performed at rest according to the guidelines of the European Society of Cardiology^66^. The public domain Kubios HRV Software 2.0 (Biosignal Analysis and Medical Imaging Group, Department of Physics and Mathematics, University of Eastern Finland, Finland) was used for data analysis. HRV-DB with calculation of the expiratory–inspiratory heart rate difference (*E*–*I* difference) and ratio (*E*–*I* ratio) were performed to characterize phasic parasympathetic activity^67^.

To investigate the impact of AF ablation on regional left ventricular sympathetic innervation, cardiac planar SPECT imaging was performed 4 h post injection of 185 MBq of MIBG (Adreview, Eindhoven, GE Healthcare B.V., The Netherlands) in patients with or without PVCs following catheter ablation of AF. Data acquisition was performed using a two-head SPECT system with standard LEHR collimators of the manufacturer (ECAM variable angle or Symbia T; Siemens Medical Solutions, Hoffman Estates, IL, USA). We used a circular 180 degrees acquisition orbit, 32 projections (64 images) with 40 s per projection (zoom 1.45, matrix 64 × 64). After reconstruction, all SPECT data were analysed using the corridor 4D-MSPECT software package (Version 5.1, INVIA Medical Imaging Solutions, Ann Arbor, MI, USA) and the dedicated normal MIBG database of the Japanese Society of Nuclear Medicine^68^. Standardized regions of interest (ROIs) for the heart were drawn under careful consideration of the left ventricular border excluding surrounding lung and gastrointestinal areas with MIBG uptake and closing the ROI at the base of the left ventricle. A mediastinal ROI of at least 4 cm^2^ in size was placed over a mediastinal region with visual minimum of regional tracer uptake. The heart to mediastinum ratio was calculated 4 h post injection. Using the 17-segment model of the American Heart Association and the dedicated normal data base of the Japanese Society of Nuclear Medicine, regional tracer uptake was analysed semiquantitatively^68^ using a summed defect score (0 for physiological and 4 for absent tracer uptake), which was calculated as the sum of all segmental defect scores.

### Statistical analysis

Continuous variables are reported as mean±s.e. of the mean (s.e.m.) and categorical variables as absolute and relative numbers. The sample size was chosen according to current state-of-the-art experimental setup and practicability^69^. No randomization was done to assign animals to experimental groups and no statistical method was used to estimate sample size for animal studies. Investigators were not formally blinded during the experiment or when assessing the outcome, but no subjective assessments were made. Normal distribution was analysed using Kolmogorov–Smirnov test. Parametric tests were not chosen when variances between the compared groups were significantly different and the number of compared values was not similar. For comparisons between groups with normally distributed data, Student's *t*-test was used for continuous variables. To compare differences across subgroups with not normally distributed data, Mann–Whitney or Wilcoxon signed-rank tests were used, as appropriate. Differences in incidences of VT occurrence were analysed using Fisher's exact test. Statistical significance was defined as a *P* value of <0.05 and is indicated in the figures. Statistical analysis was performed using Graphpad Prism 5 (Graphpad Inc., La Jolla, CA, USA).

### Data availability

The clinical study design has been registered at Clinicaltrials.gov under the identifier NCT02699255. The other data supporting the findings of this study are included in the article and its Supplementary Information files or are available from the corresponding authors upon reasonable request.

## Additional information

**How to cite this article:** Jungen, C. *et al*. Disruption of cardiac cholinergic neurons enhances susceptibility to ventricular arrhythmias. *Nat. Commun.* **8,** 14155 doi: 10.1038/ncomms14155 (2017).

**Publisher's note:** Springer Nature remains neutral with regard to jurisdictional claims in published maps and institutional affiliations.

## Supplementary Material

Supplementary Information Supplementary Figures and Supplementary Tables.
