# Supplementary material for: Disruption of cardiac cholinergic neurons enhances susceptibility to ventricular arrhythmias
Source: Nat Commun. 2017 Jan 27;8:14155. doi: 10.1038/ncomms14155 (PMC5290156; doi:10.1038/ncomms14155)
Supplement: Supplementary Information — Supplementary Figures and Supplementary Tables. [file ncomms14155-s1.pdf]

Supplementary Figures and Figure Legends

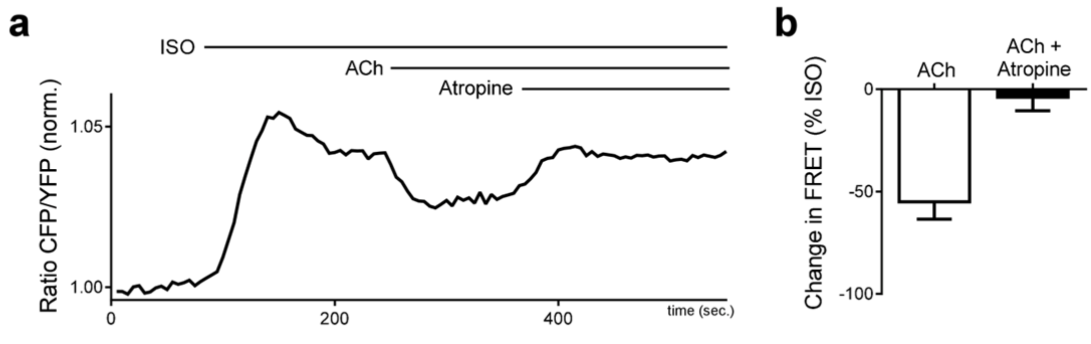

Supplementary Figure 1:

cAMP levels in myocytes confirm studies in perfused hearts.

(a) Time-resolved cAMP dynamics (presented as a normalized CFP/YFP FRET ratio) in cardiomyocytes (n = 5) after whole-cell beta-receptor stimulation (isoproterenol, ISO, $1x10^{-7}$ M), cholinergic stimulation (acetylcholine, ACh, $1x10^{-5}$ M) and muscarinergic blockade (atropine, $1x10^{-5}$ M). The black lines above the example depict the time of chemical addition. **(b)** Quantification of the data from experiments described in (a). Relative FRET changes are depicted comparing mean cAMP activity after ISO stimulation with cholinergic stimulation or muscarinergic blockade (n = 5; P=0.063; Wilcoxon signed-rank test). Data are mean ± s.e.m.

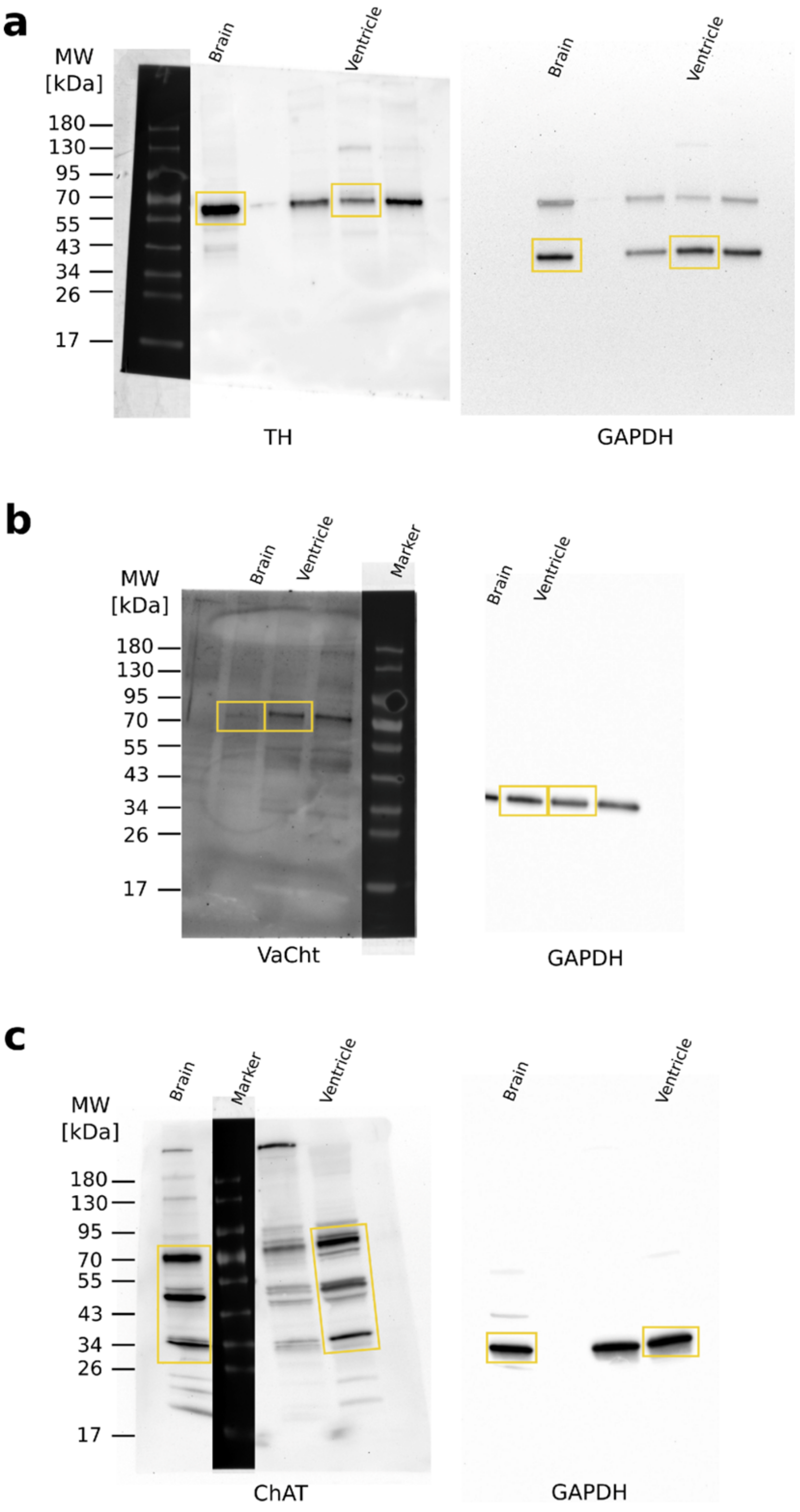

Supplementary Figure 2: Original Western blots used to generate Figure 5h.

Yellow line boxes highlight the regions of the Western blots shown in the figure.

Supplementary Tables

Supplementary Table 1: Cardiac electrophysiologic variables of murine *ex vivo* studies with effects of pharmacological modulation

	**n**	**SCL**	**SNRT$_{100}$**	**SNRTc$_{100}$**	**SNRT/SCL [%]**	**WBP**	**AVNRP$_{100}$**	**ARP$_{100}$**	**VRP$_{100}$**
**Control**									
Control	10	219±35	278±44	58.6±10.1	126.6±2.4	81±2.4	68.4±8.6	35.8±2.0	31.6±2.2
Ganglionic blockade	5	216±31	262±37	46.2±6.5	121.7±6.5	74±1.5	59.6±2.5	28.4±0.4*	22.4±1.0*
Muscarinergic blockade	5	215±42	247±45	32.0±6.9	116.3±6.9*	75±1.8	60.4±2.0	25.2±0.8*	24.4±0.7*
Beta blockade	5	199±11	276±19	76.2±11.3	138±5.2*	85±6.6	66.0±9.0	28.0±1.8*	34.8±2.4
Cholinergic stimulation	5	279±39	353±30	59.2±17.9	130±8.5	114±6.9*	100±2.6*	25.6±1.2*	35.6±1.2
**PAD**									
PAD	10	200±21	251±24	48.6±9.8	126.3±6.7	74±2.1*	58.7±2.3*	25.6±1.5*	23.4±0.9*
Ganglionic blockade	5	212±51	298±91	86.5±40.0	136±7.2	76±2.2	63.5±2.8	22.8±1.6	22.0±0.9
Muscarinergic blockade	5	158±14	202±18	43.0±12.6	128±8.5	77±2.5	63.0±2.1	27.5±2.1	24.4±0.7
Beta blockade	5	156±4	214±10	57.8±7.4	137±4.4	70±3.0	56.4±3.5	25.6±0.4	25.2±0.5
Cholinergic stimulation	5	205±9	297±8	92.0±1.2†	145.0±1.2†	129±15†	99.5±11†	33.6±2.2†	37.2±2.6†

All values are mean ± s.e.m. with intervals given in milliseconds, if not stated otherwise. ARP, atrial refractory period; AVNRP, atrioventricular node recovery period; PAD, partial atrial denervation; SCL, sinus cycle length; SNRT, sinus node recovery time; SNRTc, corrected SNRT; VRP, ventricular refractory period; WBP, wenckebach point. P<0.05, compared to control = *; compared to PAD = †.

Supplementary Table 2:

Electrocardiogram characteristics of murine *in vivo* studies

**Heart rate** [bpm]	**RR interval**	**PR interval**	**P wave duration**	**QRS duration**	**QTc**	**Tpeak-Tend**	**R Amplitude** [V]
457±26.8	135±7.5	38±1.0	20±2.4	10±0.4	46±2.1	39±2.4	1.6±0.2
All values are mean ± s.e.m. with intervals given in milliseconds, if not stated otherwise. Bpm, beats per minute; QTc = QT $(RR)^{-1/2}$.							

Supplementary Table 3:

Cardiac electrophysiological parameters of murine *in vivo* studies

**SCL**	**SNRT**	**SNRTc**	**SNRT/SCL** [%]	**WBP**	**AVNRP**	**ARP**	**VRP**
109±2	138±5	29.3±4.8	127.2±4.5	64.2±1.4	45.9±1.7	23.0±1.3	29.3±2.4
All values are mean ± s.e.m. with intervals given in milliseconds. ARP, atrial refractory period; AVNRP, atrioventricular nodal refractory period; SCL, sinus cycle length; SNRT, sinus node recovery time; SNRTc, corrected sinus node recovery time; VRP, ventricular refractory period; WBP, wenckebach point.							

Supplementary Table 4:

Transthoracal echocardiography parameters of murine *in vivo* studies

**HR** [bpm]	**LVDs** [mm]	**LVDd** [mm]	**LVPWs** [mm]	**LVPWd** [mm]	**EF** [%]	**LV FS** [%]	**CO** [ml/min]
432±14	3.87±0.2	4.90±1.1	1.44±0.4	0.99±0.2	41.6±4.0	20.5±2.3	20.0±2.2

All values are mean ± SD. CO, cardiac output; EF, ejection fraction; HR, heart rate; LVDd, diastolic left ventricular diameter; LVDs, systolic left ventricular diameter; LV FS, left ventricular fractional shortening; LVPWd, diastolic left ventricular posterior wall diameter; LVPWs, systolic left ventricular posterior wall diameter.

Supplementary Table 5: Primary antibodies

Antigen	Host	Company	Order no	Dilution WM	Dilution IHC	Dilution WB
Anti-Choline Acetyltransferase (ChAT)	Goat	EMD Millipore Cooperation, Temecula, U.S.	#AB144P	1:50	1:50	1:500
Tyrosine hydroxylase (TH)	Rabbit	EMD Millipore Cooperation, Temecula, U.S.	#AB152	1:1000	1:500	1:1000
Anti-Neurofilament H Antibody	Chicken	EMD Millipore Cooperation, Temecula, U.S.	#AB5539	1:3000	1:1000	/
Anti Vesicular Acetylcholine Transporter (VACht)	Goat	EMD Millipore Cooperation, Temecula, U.S.	#abn100	/	/	1:500
HRP-conjugated anti GAPDH 14C10	Rabbit	Cell Signaling Technology Danvers, MA, US	#3683	/	/	1:2000

Supplementary Table 6: Secondary antibodies

Antigen	Host	Company	Order no	Dilution WM / IHC
Alexa Fluor 488 anti-rabbit IgG	Donkey	Thermo Fisher Scientific, Waltham, USA	A-21206	1:500
Alexa Fluor 488 anti chicken IgY	Goat	Thermo Fisher Scientific, Waltham, USA	A-11039	1:500
Alexa Fluor 568 anti-goat IgG	Donkey	Thermo Fisher Scientific, Waltham, USA	A-11057	1:500
Alexa Fluor 647 anti-chicken IgY	Donkey	Thermo Fisher Scientific, Waltham, USA	AP194SA6	1:500
Anti-Rabbit IgG biotin-conjugated	Donkey	EMD Millipore Cooperation, Temecula, USA	AP182B	1:200
Anti-Goat IgG biotin-conjugated	Donkey	R&D Systems Inc., Minneapolis, USA	BAF109	1:200
Anti-Chicken IgY biotin-conjugated	Goat	R&D Systems Inc., Minneapolis, USA	BAF010	1:200
Anti-Goat IgG, POX-conjugated	Horse	Vector Laboratories, Burlingame, USA	PI-9500	1:10.000

Supplementary Table 7: Patient characteristics

	**No PVC** (n=105)	**PVC** (n=6)	***P*** **-** Value
Age [years]	60±1	66±3	0.14
Male	64 (64)	3 (50)	0.68
Body mass index [kg m^{-2}]	27±0.4	26±1.7	0.64
LVEF [%]	61±0.6	62±1.3	0.46
LA size [mm]	39±0.6	37±1.9	0.41
**Medical Treatment**			
Amiodarone	8 (8)	2 (33)	0.65
Class I antiarrhythmic drugs	38 (36)	2 (33)	0.67
Beta-receptor blocker	69 (66)	5 (83)	1.0
ACE / ARB	30 (29)	4 (67)	0.19
Diuretics	11 (10)	1 (17)	1.0

Values are mean ± s.e.m. or n (%) if not stated otherwise. ACE, angiotensin converting enzyme inhibitor; ARB, angiotensin II receptor blocker; LA, left atrial; LVEF, left ventricular ejection fraction; No PVC, asymptomatic patients or patients without an increased PVC burden after catheter ablation for atrial fibrillation.

Supplementary Table 8: Autonomic characterization of patients before and after PVI

	**Before PVI** (n=10)	**After PVI** (n=10)	***P* -Value**
**Heart Rate Variability**			
Time-domain			
SDNN [ms]	31.2±2.9	13.9±3.2	0.006
RMSSD [ms]	17.4±2.0	8.2±1.9	0.002
Frequency-domain			
LF (AR) [ms^2]	327.1±65.5	63.1±33.5	0.002
HF (AR) [ms^2]	117.4±24.9	45.7±24	0.049
LF/HF (AR)	3.2±0.7	1.2±0.4	0.049
Non-linear			
SD_1 [ms]	12.3±1.4	5.8±1.4	0.002
REC [%]	34.1±2.8	38.8±3.8	0.408
DFA_1	1.3±0.04	1.0±0.1	0.039
**Deep Breathing**			
E-I Differenz [min^{-1}]	7.3±1.2	3.5±0.6	0.006
E/I Ratio (Norm: ≥ 1.1)	1.1±0.03	1.0±0.008	0.004
**Ventricular Repolarization Heterogeneity**			
QT dispersion [ms]	29.5±3.7	29.3±3.9	0.971

All values are mean ± s.e.m.. SDNN, standard deviation of normal to normal R-R intervals; SD_1, short-term RRI variability; LF, low frequency; HF, high frequency; REC, recurrence rate; DFA, detrended fluctuation analysis; RMSSD, root mean square of successive heartbeat interval differences; E, expiration; I, inspiration.
